# Sex and Genetic Differences in Behavioral Engagement of Crossed High Alcohol‐Preferring and Low Alcohol‐Preferring Mice

**DOI:** 10.1111/gbb.70026

**Published:** 2025-06-10

**Authors:** Phillip Starski, Addyson Siegle, Danielle White, Bea Paras, Christy Tham, Maribel Hernandez, Alecsander Zareb, Nicholas Grahame, Stephen L. Boehm, Frederic Hopf

**Affiliations:** ^1^ Dept. Psychiatry Indiana University School of Medicine Indianapolis Indiana USA; ^2^ Dept. Psychology Indiana University‐Indianapolis Indianapolis Indiana USA

**Keywords:** 5‐choice, 5‐CSRTT, alcohol, alcohol preference, attention, cHAP, impulsivity, LAP, perseveration, sex differences

## Abstract

Excessive levels of alcohol consumption play a major role in numerous alcohol‐related harms, including a heightened risk of developing problematic drinking behaviors. Those who develop alcohol use disorder (AUD) often struggle with persistent difficulties in controlling their drinking, experience withdrawal symptoms, and engage in risky behaviors that pose danger to themselves and others. Advances in treating AUD may be supported by identifying specific cognitive and emotional factors that drive excessive alcohol consumption. Recognizing reliable behavioral biomarkers is instrumental in assessing the risk of developing alcohol problems and preventative care strategies. This study investigates innate behavioral differences associated with genetic predisposition for alcohol use by comparing crossed high alcohol‐preferring (cHAP) and low alcohol‐preferring (LAP) mice. Since there have been links between heightened impulsivity and excessive alcohol use, we hypothesized that cHAP mice would exhibit higher levels of impulsivity compared to LAPs. No significant differences were found in impulsivity between the mouse lines or sexes. cHAPs adapted to shorter stimulus durations (SDs), whereas LAPs showed a marked decline in correct responses and an increase in omission rates as task difficulty increased. Significant sex differences within the cHAP line were found, with females demonstrating higher accuracy, lower correct latency, and increased perseveration. This behavior points to potential sex‐specific neural activation in cognitive processing areas. Future studies should explore salient brain regions to understand their roles in behavioral regulation and sex‐specific responses to challenges. This study provides a foundation for exploring the interaction of genetic predisposition, sex differences, and neural mechanisms in alcohol preference and behavior.

## Graphical abstract

This study reveals genetic and sex‐specific behavioral differences between crossed high alcohol‐preferring (cHAP) and low alcohol‐preferring (LAP) mice. cHAPs showed greater adaptability to task difficulty, while LAPs exhibited increased omission rates. Notably, cHAP females demonstrated higher accuracy and perseveration, suggesting potential sex‐specific neural mechanisms to target alcohol‐related behavior and cognitive processing.

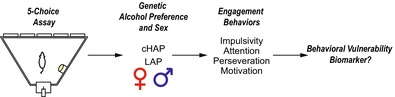

**Abbreviations**
5‐Choice: 5‐choice serial reaction time task
cHAP: crossed high alcohol‐preference
LAP: low alcohol‐preference

## 1 Introduction

Excessive alcohol consumption significantly contributes to a range of alcohol‐related harms, including an increased risk of developing drinking problems [1, 2]. Individuals who go on to develop alcohol use disorder (AUD) experience a chronic inability to control their drinking, face withdrawal symptoms, and may engage in risky behaviors that harm themselves and others [3, 4, 5]. Recent findings have shown that these effects can be magnified in vulnerable populations, such as women, who are increasingly consuming more alcohol across age groups and may face a higher risk for severe alcohol‐related issues [6, 7, 8, 9, 10]. Future AUD treatment may benefit from identifying specific cognitive and emotional factors that drive excessive drinking. Thus, identifying reliable behavioral biomarkers to assess the risk of developing alcohol problems could provide valuable insights for preventative care.

The DSM‐V outlines behavioral patterns related to alcohol acquisition and intake and uses their frequency and severity to determine the severity of AUD in individuals. Importantly, these behaviors come from natural, everyday actions, such as attentiveness toward a crying child, motivation toward personal goals, or acting impulsively in urgent situations. Here, we refer to these as “engagement behaviors.” While typically adaptive, when these behaviors occur more frequently or in maladaptive forms, they are associated with a heightened risk of developing AUD. For instance, studies have demonstrated a strong connection between excessive alcohol use and impulsivity, especially during early drinking behaviors, such as the first drink, the initiation of binge‐drinking episodes, and throughout other stages of AUD progression [11]. Recent studies have demonstrated further that a higher risk of alcohol binging has been linked to high trait impulsivity [12, 13, 14], which is also associated with greater alcohol intake and subjective intoxication [15, 16]. AUD is often accompanied by motivational changes, including an intensified desire for intoxication and an increased tolerance for adverse consequences [17, 18, 19]. Furthermore, an “attentional bias” often develops that promotes behavior oriented toward alcohol‐related cues [20, 21, 22]. Attention to alcohol‐related cues plays a critical role in the development of AUD, with studies showing heightened activation in brain regions such as the frontoparietal network [23] and the salience network, particularly the anterior cingulate cortex and insula [24]. Therefore, assessing engagement behaviors may be essential for predicting risk, informing prevention strategies, and guiding treatment for AUD.

Individuals with a family history of AUD have approximately a 50% chance of inheriting the disorder and having an earlier onset and more serious health problems [25]. There is an ongoing effort in alcohol use research to establish a genetic framework for the heritability of traits associated with AUD. Given the broad range of behaviors involved in alcohol‐seeking and consumption, there is a growing need to identify more specific behavioral endophenotypes that can be reliably linked to genetic risk. One promising approach for modeling AUD with a familial component in the laboratory is through the use of selectively bred crossed high alcohol‐preferring (cHAP) mice. These mice were created by crossing two independently bred lines, HAP replicate 1 (HAP1) and HAP replicate 2 (HAP2), which were selectively bred from the HS/Ibg background for high voluntary intake during a 4‐week, free‐choice 10% ethanol paradigm [26, 27]. cHAP mice may reach over 250 mg/dL during continuous voluntary access paradigms and will display compulsive‐like drinking by overcoming quinine‐adulteration [28, 29, 30]. Conversely, the low alcohol‐preferring (LAP) mice were selectively bred in a similar manner but for minimal alcohol consumption, consistently drinking little to no alcohol. While the HAP replicate lines were crossed to potentially enhance alcohol intake in the offspring, the LAP replicate lines were not crossed, as further reducing an already extremely low level of intake was unlikely to produce meaningful differences from the parent lines. Interestingly, quantitative trait locus analysis identified genomic region differences between HAP and LAP replicate lines. Notably, reduced expression of *Gnb1*, a candidate gene, enhances alcohol intake by diminishing taste or smell aversion, rather than by increasing the rewarding effects of alcohol [31]. Further differences driven by alcohol preference were found on the mid region of chromosome 9 with suggested candidate genes *Drd2*, which encodes the dopamine D2 receptor involved in the reward effects of alcohol [32, 33], and the 5‐HT_1B_—serotonin receptor encoder *Htr1b*, known to be involved in alcoholism with antisocial impulsivity [34, 35, 36, 37]. Unlike other mouse models of excessive alcohol intake, such as equilibrative nucleoside transporter‐1 knockout mice [38], these selectively bred heterogeneous lines have greater face validity when investigating a family history. Together, the cHAP and LAP lines provide a more translational model for studying the role of family history in alcohol use, capturing both high and low heritable risk profiles.

Replicating the complex behavioral endophenotypes observed in patients with AUD within an animal model requires careful consideration of specific engagement behaviors [39]. To address this challenge, we have taken a targeted approach by dissecting distinct behavioral components across the various stages of the 5‐Choice Serial Reaction Time Task (5‐Choice), with a focus on how these behaviors shift in response to increased task demands. Behavioral engagement encompasses a range of cognitive and motivational processes, many of which have been individually examined in separate paradigms [40, 41, 42]. The 5‐Choice is a widely used assay, since one can assess different aspects of behavioral engagement in the same session, through indicators of motivation (e.g., tray entries, reward latency), attention (e.g., accuracy, omissions), perseveration (e.g., repeated responses), and impulsivity (e.g., responses during waiting period) [43, 44, 45, 46, 47, 48, 49, 50]. The establishment of behavioral endophenotypes requires that they be independent of disease state. In the current study, we assessed alcohol‐naive cHAP and LAP mice in the 5‐Choice task to determine whether genetic selection for alcohol preference alone changes innate behavioral engagement. In rodent‐preference models, previous work has shown greater impulsivity in HAP1 and HAP2 mice compared to LAP mice in delay discounting tasks [51, 52]; however, unlike delay discounting, which reflects cognitive impulsivity through the devaluation of delayed rewards [53, 54, 55], the present study measures waiting impulsivity, a more motor‐based, anticipatory response form of impulsivity [56, 57, 58, 59]. Further, perseveration, characterized by the inability to disengage from previously rewarded behaviors [60, 61] (i.e., compulsive‐like behavior), has not been observed in attentional set‐shifting tasks with cHAP and LAP mice [62], although cHAP mice with alcohol exposure have developed habitual behaviors in operant devaluation tasks [28]. Individuals with a family history of AUD tend to be more sensitive to alcohol and high‐sweet taste cues [63, 64], but innate attention and further motivational maladaptation in cHAP mice have not yet been explored. We hypothesized that mice with greater genetic vulnerability to alcohol use, reflected by a family history of AUD, would display distinct changes in engagement behaviors as task demands escalate in the 5‐Choice. Specifically, we predicted heightened impulsivity, motivation, and attention in these mice, particularly in response to a highly palatable sweet reward, which may serve as an early behavioral marker of risk for developing AUD.

## 2 Materials and Methods

### 2.1 Animals

Forty cHAP mice (cHAP, 20 M, 20F 53rd generation) and 20 LAP mice (10M, 10F, 51st generation). All mice were born in the Indiana University‐Indianapolis Animal Care Facilities. All mice were individually housed for 1 week before the start of the experiment in a 12:12 h reverse light–dark cycle (7:00: off, 19:00: on) at 56–61 days of age. Mice were housed in standard Plexiglass cages with *ad libitum* access to food and water until food restriction. Behavioral sessions occurred between 8:00 and 14:00, during the dark phase of their cycle. Animal care and handling procedures were approved by the Indiana University Institutional Animal Care and Use Committee in accordance with National Institutes of Health guidelines.

### 2.2 5‐Choice Serial Reaction Time Task (5‐Choice)

*Apparatus and food restriction:* Eight noise‐attenuating Bussey‐Saksida Rodent Touch Screen Chambers (Lafayette Instruments Co., Lafayette, IN) were used to assess 5‐Choice behavior. Each chamber had perforated stainless‐steel floors with trapezoidal plastic walls enclosed with a touchscreen (12.1in, resolution 800x600), 3 W lights, a speaker (65 dB tone), ventilation fans, and an infrared sensitive camera. A plastic mask outlining five 4x4 cm holes spaced 1 cm apart and 1.5 cm from the floor was used to cover each screen. Food was given immediately after the behavioral session. Animals were food restricted to no less than 85% of their *ad libitum* weight. Importantly, LAP mice were given ~2 g of chow daily after 5‐Choice behavior, while cHAP mice were given ~3‐4 g of chow. cHAPs were initially given ~2 g to stabilize body weight but would become severely lethargic the next day. cHAPs displayed greater overall activity, qualitatively, within their home cage and during the behavioral task (Video 1: cHAP vs. Video 2: LAP).

#### 2.2.1 Pre‐Training

Mice experienced two habituation days for 15 min, where 20ul of strawberry milk was delivered after every tray exit to familiarize the mice with the reward location. The next 3 days used a “Must‐Touch” Fixed‐Ratio 1 schedule, where one of the stimulus holes illuminated until it was touched. Following a correct touch, 20 μL of strawberry milk was delivered, and the illuminated hole changed to a different location. These sessions lasted 60 min, with an unlimited number of trials.

#### 2.2.2 Training

After pretraining, mice followed 5‐Choice training (Table 1). Mice were placed into the dark operant chamber and 200ul of strawberry milk was delivered under a light illuminated in the reward tray as a free reward. Once their head left the reward tray, the first trial was initiated. A 2 s intertrial interval (ITI), or waiting period, occurs before one of the five holes is illuminated for 20 s. At this point, three different scenarios can occur. 1) A correct touch into the illuminated hole occurs, which triggers delivery of 20 μL strawberry milk into the reward tray. 2) An incorrect touch into one of the unilluminated holes results in a punishing flash and tone. 3) If there is no touch during the SD or the 5 s grace period (Limited Hold, LH) resulting in an omission, it receives a punishing flash of light and tone. In the event of an incorrect response or omission, the reward tray illuminates and requires a head entrance to initiate the next trial. Additionally, if there is a touch during the ITI, this results in a premature response and restarts the ITI timer. Touches after a correct or incorrect response into one of the stimulus holes are recorded as a perseverative response. Table 1 (Stage 1–5) details the duration and progress of the training schedule.

**TABLE 1:** Behavior Experimental Schedule.

Stage	Stimululs duration (s)	Intertrial interval (s)	Days	Test	Analysis
1	20	5	10	Training	—
2	10	5	10	Training	—
3	5	5	10	Training	Figure 1; S‐S3
4	2	5	5	Training	Figure 1; S‐S3
5	1	5	5	Training	—
6	1	5	5	Baseline	Figures 1, 3; S‐S3, S7
**7**	**1**	**Randomized**	**5**	**Impulsivity**	Figure 3 **; S7**
8	5	5	5	Baseline	Figures 1, 2; S1‐S6
**9**	**5**	**Randomized**	**5**	**Impulsivity**	Figure 2 **; S4‐S6**
**10**	**Randomized**	**5**	**5**	**Attention**	Figures 2, 3 **; S7**

*Note:* Bold stages represent testing sessions. Stimulus duration is the time the light in one of the five stimulus ports is on. After, there is a five second limited hold period. Intertrial interval is the waiting period before the stimulus presents itself. Impulsivity tests randomized the waiting period (ITI) between 2, 5, 7, 10, 15 s. Attention tests randomized the stimulus duration between (0.5, 1, 1.5, 2, 2.5 s).

#### 2.2.3 Baseline, Impulsivity, and Attention Testing

Once mice reached a 1 s SD, an additional 5 days were recorded to serve as a baseline used in subsequent analyses. To test impulsivity between cHAP and LAP mice, 5 days of 5‐Choice behavior where the ITI was randomized (2, 5, 7, 10, 15 s) to challenge their ability to wait. Interestingly, after 1 s SD baseline and impulsivity testing, LAP mice displayed consistently poor performance compared to cHAP mice and made any secondary comparisons (premature, perseverative, etc.) between the lines less meaningful. Thus, all mice were backtracked to a 5 s SD where cHAP and LAPs were more comparable in the number of correct responses. Five days of baseline testing were recorded before performing impulsivity testing at a 5 s SD. This greatly increased the performance of the LAP and allowed better comparison to cHAP mice. Lastly, attention was challenged in the mice with 5 days where the ITI had remained static at 5 s and the SD was randomized (0.5, 1, 1.5, 2, 2.5 s).

#### 2.2.4 Exclusion Criteria

**FIGURE 1:**
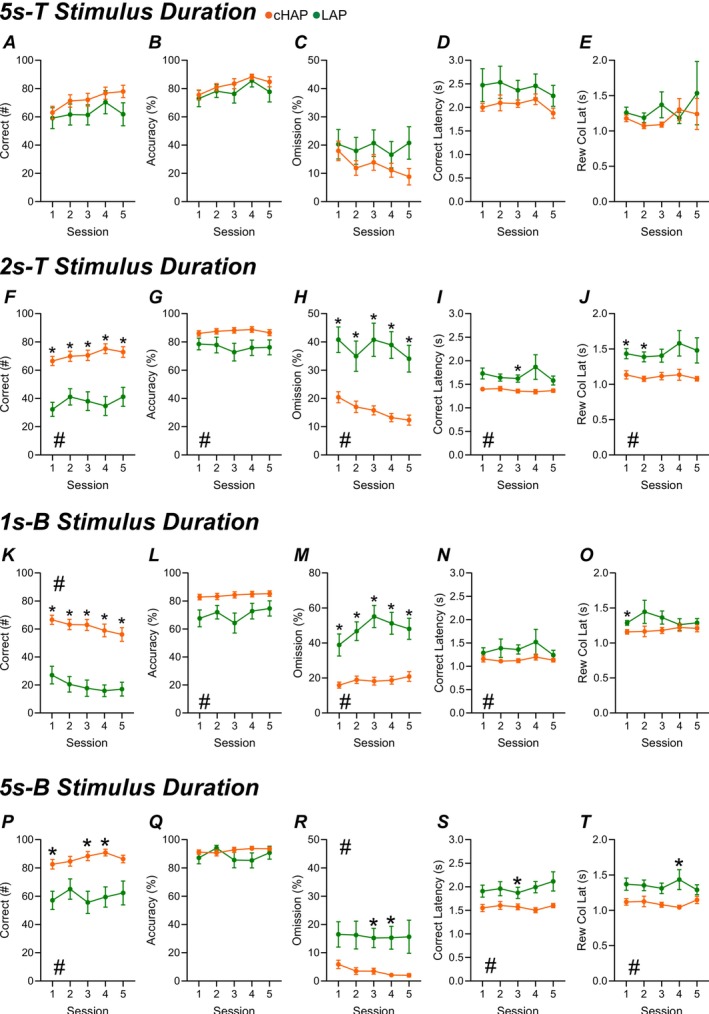
Genetic Alcohol Preference Effect on 5‐Choice Performance Progression. cHAP (orange) and LAP (green) lines were compared during the following stimulus training levels: Five second training (5 s‐T), two second training (2 s‐T), one second baseline (1 s‐B), and the re‐established five second baseline (5 s‐B). **A, F, K, P**) Correct responses into the touch port at the respective stimulus. **B, G, L, Q**) Overall accuracy of responses (correct/[correct+incorrect]*100). **C, H, M, R**) Percentage of trial omissions (omitted trials/100*100). **D, I, N, S**) Time to give a correct response. **E, J, Q, T**) Time to retrieve the reward. This analysis did not exclude any mice to demonstrate variability within the mouse lines. cHAP (*n* = 20/sex), LAP (*n* = 10/sex). Error bars represent standard error of the mean. *p < 0.05.

**FIGURE 2:**
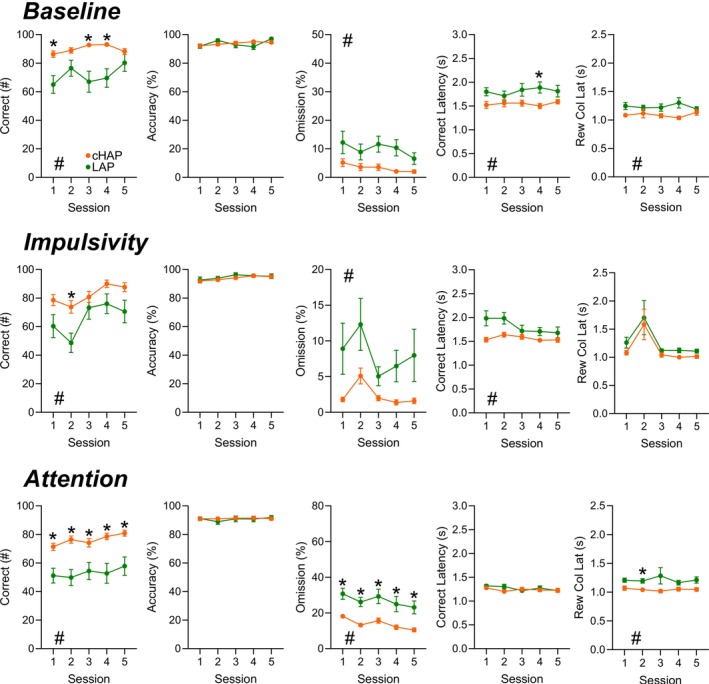
Genetic Preference Effect on 5‐Choice Challenge Tests. cHAP (orange) and LAP (green) mice established a baseline performance under a 5 s stimulus duration and 5 s waiting period (Top). Impulsivity testing, where the waiting period was randomized (2, 5, 7, 10, 15 s), was performed with a 5 s stimulus duration (Middle). Attention testing was performed by randomizing the stimulus duration (0.5, 1, 1.5, 2, 2.5 s) and having a static waiting period of 5 s (Bottom). **A, F, K**) Correct responses. **B, G, L**) Overall Accuracy. **C, H, M**) Omission percentage. **D, I, N**) Correct latency. **E, J, O**) Reward collection latency. Error bars represent standard error of the mean. *p < 0.05.

**FIGURE 3:**
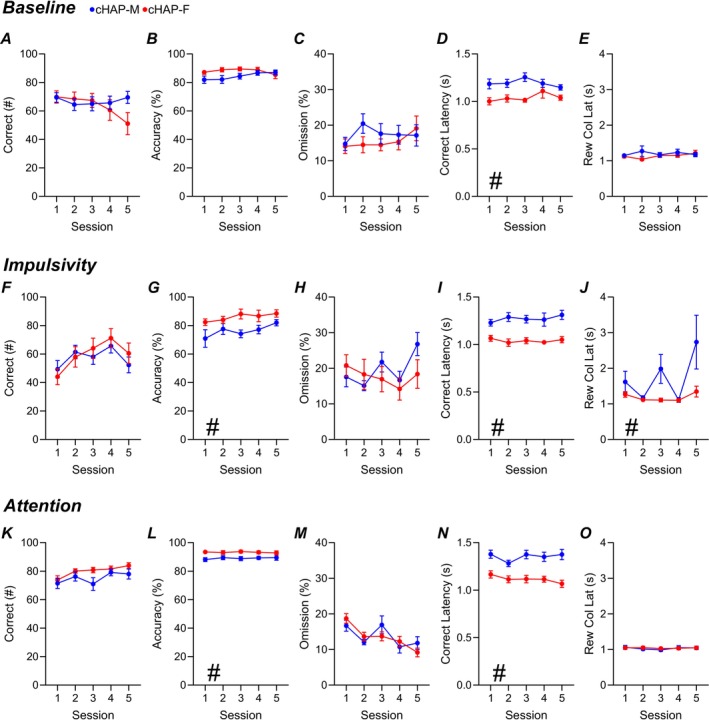
Performance Differences Between cHAP Males and Females. cHAP mice established a baseline performance under a 1 s stimulus duration and 5 s waiting period (Top). Impulsivity testing was performed with a 1 s stimulus duration (Middle). Attention testing was performed by randomizing the stimulus duration and having a static waiting period of 5 s (Bottom). **A, F, K**) Correct responses. **B, G, L**) Overall Accuracy. **C, H, M**) Omission percentage. **D, I, N**) Correct latency. **E, J, O**) Reward collection latency. cHAP‐Males (blue, *n* = 19), cHAP‐Females (red, *n* = 19). Error bars represent standard error of the mean. *p < 0.05.

All mice were (Tables S1–S5) included in Figure 1 to demonstrate the range in behavior seen in cHAP and LAP mice regardless of performance. In Figures 2 and 3, mice that did not average more than 20 correct responses across the five sessions of 5 s Baseline (5 s‐B) were not included in impulsivity and attention analyses. This includes two male LAP and one male cHAP mouse. One female cHAP died unexpectedly during attention testing and has been excluded from impulsivity and attention analyses. Additionally, errant sessions of individual mice that differ greatly were excluded from analysis. These sessions typically result in extremely low correct responding (less than 10 compared to greater than 50) and may stem from experimenter error or animal temperament. This resulted in two sessions of two separate female LAP mice and one session of a single male cHAP mouse during 5 s‐B (295 sessions total, 3 excluded sessions). In Figure 3, one cHAP male mouse and three cHAP female mice were completely excluded from analysis due to low responding (< 11 correct responses). Lastly, in Figure 3, individual sessions were excluded if a mouse gave less than 10 correct responses; the four other sessions were averaged. This results in one male and one female session during 1 s‐baseline and three total sessions from different female mice and two sessions in different male mice during 1 s‐impulsivity testing.

### 2.3 Statistical Analysis

Behavioral data from the 5‐Choice task were analyzed by two‐way repeated measures analysis of variance (ANOVA) followed by Bonferroni's multiple comparisons test where appropriate. Sphericity was not assumed, and the Geisser–Greenhouse correction was used. For missing data points, a mixed‐model analysis of variance was used. All statistical analyses were performed using Prism 9.0 (GraphPad Software Inc., San Diego, CA), with statistical significance defined as *p* < 0.05.

## 3 Results

### 3.1 LAP Mice Decrease Performance as Task Difficulty Increases

Innate alcohol preference may serve as an important risk factor in the development of AUD. Here, we seek to characterize other innate behaviors of genetically predisposed HAP and LAP mice that may influence AUD development. All mice were initially trained in the 5‐Choice Serial Reaction Time Task (5‐Choice) to reach a 1‐s stimulus duration (SD) baseline (Table 1). Performance across training stages (5 s‐T and 2 s‐T SD) and baseline sessions (1 s SD [1 s‐B] and 5 s SD baseline [5 s‐B]) is summarized in Figure 1. All animals were included to examine comprehensive line differences in learning and task performance.

Notable differences were seen during the training stages between cHAP and LAP mice. During the 5 s SD training phase, the only significant mouse line difference was in perseverative responding (Figure S1D), with cHAP mice displaying elevated levels. At the 2 s SD training phase, cHAP mice exhibited significantly more initiated trials (Figure S1F), greater numbers of correct responses (Figure 1F), and higher accuracy (Figure 1G), alongside significantly lower omission rates (Figure 1H), shorter latencies to respond correctly (Figure 1I) or incorrectly (Figure S1G), and faster reward collection latencies (Figure 1J). These patterns largely persisted into the 1 s SD baseline phase (Figure 1K–O), although reward collection latency no longer differed between groups (Figure 1O). Additionally, LAP mice began to show significantly higher premature response rates (Figure S1M). During the 5 s‐B sessions, cHAP mice maintained significantly greater trial counts (Figure S1P) and correct responses (Figure 1P), with lower omission rates (Figure 1R) and reduced latencies to correct (Figure 1S) and reward collection (Figure 1T). All statistical information is outlined in Table S1.

### 3.2 cHAP Mice Exhibit Enhanced Task Performance Compared to LAP Mice, With Distinct Sex‐Specific Patterns

To explore potential sex‐dependent effects that may drive the omnibus differences between the lines, we conducted additional analyses comparing performance by sex (e.g., cHAP females vs. LAP females, cHAP males vs. LAP males). During 5 s SD training, cHAP males showed significantly more correct responses (Figure S2A), lower omission rates, and faster reward collection (Figure S2E), correct, and incorrect response latencies (Figure S2D, Figure S3B) compared to LAP males. In contrast, cHAP females only differed from LAP females in perseverative responding (Figure S3D). At the 2 s SD training stage, cHAP males exhibited significantly higher accuracy (Figure S2G), fewer omissions (Figure S2H), and faster reward collection and correct response latencies (Figure S2J, Figure S2I) compared to LAP males. Among females, cHAP mice outperformed LAP mice across nearly all metrics, including total trials (Figure S3F), correct responses (Figure S2F), accuracy (Figure S2G), omission rates (Figure S2H), and both reward collection and correct latencies (Figure S2J, Figure S2I). These sex‐based performance differences persisted into the 1 s SD baseline. Both cHAP males and females completed more trials (Figure S3K) and correct responses (Figure S2K) and exhibited higher accuracy (Figure S2L), and fewer omissions (Figure S2M) compared to their LAP counterparts. cHAP females also had lower correct and incorrect (Figure S2N, Figure S3L) latencies, while LAP females displayed higher premature responding (Figure S3M). During the 5 s‐B session, both male and female cHAP mice demonstrated a greater number of trials (Figure S3P) and correct responses (Figure S2P), with lower omission rates (Figure S2R), reward collection, and correct latencies (Figure S2T, Figure S2S). Additionally, cHAP females had faster incorrect latencies relative (Figure S3Q) to LAP females, while cHAP males had more ITI tray entries (Figure S3T) than LAP males. All statistical information is outlined in Table S2.

We then examined sex differences within each line (e.g., cHAP males vs. cHAP females, LAP males vs. LAP females) to assess how selective breeding for alcohol preference affected males versus females. During 5 s SD training, no significant sex differences were observed within either line. However, during the 2 s SD phase, both lines showed significantly higher perseveration in females (Figure S3I). Additionally, cHAP males had more ITI tray entries than females (Figure S3J). At the 1 s SD baseline, LAP males and females differed in trial counts (Figure S3K), while cHAP females exhibited lower correct latencies (Figure S2N) and greater perseveration (Figure S3N) than males. During the 5 s‐B session, cHAP females demonstrated greater accuracy (Figure S2Q), fewer omissions (Figure S2R), shorter correct and incorrect latencies (Figure S2S, Figure S3Q), more perseverative responding (Figure S3S), and fewer ITI tray entries (Figure S3T) compared to cHAP males. LAP females showed faster correct and incorrect latencies (Figure S2S, Figure S3Q) and more perseverative responses (Figure S3S) compared to LAP males. All statistical information is outlined in Table S2.

### 3.3 HAP Mice Display Greater Overall Performance and Lower Levels of Impulsivity Than LAP Mice

Since LAP mice showed significant performance declines at shorter SDs, comparisons of experimental measures, such as impulsivity and attention, are less meaningful when contrasted with the high‐performing cHAP mice. Thus, all mice re‐established a baseline at 5 s, a point during training where cHAP and LAPs were most similar in the number of correct trials. Compared to their 5 s‐T sessions, performance during the 5 s‐B sessions showed notable improvements in both groups, including shorter latencies, reduced premature responding, and increased accuracy. These changes suggest that both cHAP and LAP mice became more efficient in the task with additional training sessions. With this more normalized baseline between the cHAP and LAPs, impulsivity and attention testing was performed to identify any differences between the genetic lines. During these analyses, we excluded the lower performers from each group to compare the mice during cognitively challenging variations of the task.

During the 5‐s SD baseline session, cHAP mice completed significantly more trials (Figure S4A) and correct responses (Figure 2A) compared to LAP mice. They also exhibited lower omission rates (Figure 2C), shorter reward collection latencies (Figure 2E), and faster correct response latencies (Figure 2D). This pattern persisted during the impulsivity testing phase, where cHAPs again demonstrated a greater number of trials (Figure S4F) and correct responses (Figure 2F), along with lower omission rates (Figure 2H) and reduced correct response latencies (Figure 2I). Notably, LAP mice showed a trend toward increased premature responding (Figure S4H) during this phase. During attention testing, cHAP mice continued to outperform LAPs in trials completed (Figure S4K) and correct responses (Figure 2S), while also showing lower omission rates (Figure 2M) and shorter reward collection latencies (Figure 2O). Unlike in the impulsivity phase, however, the percentage of premature responses (Figure S4M) was significantly higher in LAP mice compared to cHAPs. All statistical information is outlined in Table S3.

### 3.4 Female HAP Mice Drive the Major Behavior Differences Between Lines

Analysis of sex‐specific performance within each line during these cognitive challenges revealed additional differences. During 5 s‐B sessions, cHAP females initiated more trials (Figure S6A) and correct responses (Figure S5A), had fewer omissions (**Figure 5C**), and showed faster correct and incorrect latencies (Figure 5D, Figure S6B), with a trend toward faster reward collection latency (Figure S5E) compared to LAP females. cHAP males also initiated more trials (Figure S6A) and correct responses (Figure S5A) and had fewer omissions (Figure S5C), with a trend toward shorter correct response latency (**Figure 5D**) than LAP males. During impulsivity testing, cHAP females again had more trials (Figure S6F) and correct responses (Figure S5F), fewer omissions (Figure S5H), and trends toward faster reward collection (**Figure 5 J**) and correct response latencies (Figure S5I) compared to LAP females. Notably, cHAP females exhibited lower premature responding (Figure S6H). Among males, cHAPs showed significantly faster correct latencies (Figure S5I) and trended toward fewer omissions (Figure S5H) relative to LAPs. During attention testing, cHAP females outperformed LAP females across most measures, including trial counts (Figure S6K), correct responses (Figure S5K), omission rates (Figure S5M), and reward collection latency (Figure S5O), while also showing a trend toward faster correct latency (Figure S5N). Again, premature responding (Figure S6M) was significantly lower in cHAP females. Among males, cHAPs had more correct responses (Figure S5K), fewer omissions (Figure S5M), and trended toward faster correct response latency (Figure S5N) compared to LAPs. All statistical information is outlined in Table S4.

Finally, we examined sex differences within each line during these task phases. In the 5 s‐B session, cHAP females demonstrated more correct responses (Figure S5A), greater accuracy (Figure S5B), and increased perseverative responding (Figure S6D), along with lower omission rates (Figure S5C), shorter correct and incorrect latencies (Figure S5D, Figure S6B) and fewer ITI tray entries (Figure S6E) relative to cHAP males. LAP females showed trends toward higher accuracy (Figure S5B) and lower omissions (Figure S5C), along with significantly shorter correct and incorrect latencies (Figure S5D, Figure S6B) compared to LAP males. During impulsivity testing, cHAP females exhibited higher accuracy (Figure S5G) and perseverative responding (Figure S6I), fewer omissions (Figure S5M), and shorter correct latencies (Figure S5I) compared to males. They also trended toward fewer ITI tray entries (Figure S6J). In contrast, LAP females only differed from LAP males in total trial count (Figure S6F). Similarly, during attention testing, cHAP females showed higher accuracy (Figure S5L) and perseveration (Figure S6N) and shorter correct latencies (Figure S5N) than males, while LAP females again only exceeded males in trial numbers (Figure S6K). All statistical information is outlined in Table S4. A summary of the significant behavioral differences between genetic lines can be found in Table 2 and sex differences within lines in Table 3.

**TABLE 2:** Mouse Line Differences.

	cHAP vs. LAP			cHAP vs. LAP‐females			cHAP vs. LAP‐females		
	Baseline	Impulsivity	Attention	Baseline	Impulsivity	Attention	Baseline	Impulsivity	Attention
Trials	**C>L**	**C>L**	**C>L**	**C>L**	**C>L**	**C>L**	**C>L**	—	—
Correct	**C>L**	**C>L**	**C>L**	**C>L**	**C>L**	**C>L**	**C>L**	—	**C>L**
Accuracy	—	—	—	—	—	—	—	—	
Omission (%)	**C<L**	**C<L**	**C<L**	**C<L**	**C<L**	**C<L**	**C<L**	C<L&	**C<L**
Correct Lat	**C<L**	**C<L**	—	**C<L**	C<L&	C<L&	C<L&	**C<L**	**C<L**
Incorrect Lat	**C<L**	—	—	**C<L**	—	—	—	—	—
Reward Lat	**C<L**	—	**C<L**	C<L&	C<L&	**C<L**	C<L&	**C<L**	—
Premature (%)	—	C<L&	C<L&	—	C<L&	**C<L**		C<L&	—
Perseverative (%)	—	—	—	—	—	—	—	—	—
ITI Tray Entries	—	—	—	—	—	—	—	—	—

*Note:* C = cHAP, L = LAP. Bold = *p < 0.05. ^&^Denotes *p* < 0.085.

**TABLE 3:** Mouse Line Sex Differences.

	cHAP			LAP		
	Baseline	Impulsivity	Attention	Baseline	Impulsivity	Attention
Trials	—	—	—	—	**F>M**	**F>M**
Correct	**F>M**	—	—	—	—	—
Accuracy	**F>M**	**F>M**	**F>M**	F>M&	—	—
Omission (%)	F>M&	**F>M**	—	**F>M**	—	—
Correct Lat	**F<M**	**F<M**	**F<M**	**F<M**		**F<M**
Incorrect Lat	**F<M**	—	—	**F<M**	—	—
Reward Lat	—	—	—	—	—	—
Premature (%)	—	—	—	—	—	—
Perseverative (%)	**F>M**	**F>M**	**F>M**	**F>M**		**F>M**
ITI Tray Entries	**F<M**	F<M&	—	—	—	—

*Note:* F = Female. M = Male. Bold = *p < 0.05. ^&^Denotes *p* < 0.085.

### 3.5 HAP Females Are More Attentive and Display More Compulsive‐Like Behavior

As cHAP mice were able to perform the task at a 1 s SD, we further compared sex differences within this line. During 1 s SD baseline, cHAP females had faster correct latencies (Figure 3D) and greater perseveration (Figure S7D) than males. During 1 s SD impulsivity testing, cHAP females demonstrated higher accuracy (Figure 3G), faster correct latency (Figure 3I), and a trend toward faster reward collection latency (Figure 3J). These effects remained consistent in attention testing, as previously described, with cHAP females showing greater accuracy (Figure 3L), increased perseveration (Figure S7N), and lower correct response latency (Figure 3N) compared to males. All statistical information is outlined in Table S5.

Summary results of mouse line differences are described in Table 2 and sex differences within mouse line are in Table 3.

## 4 Discussion

In this study, we aimed to investigate innate behavioral differences associated with a genetic predisposition toward alcohol use. cHAP mice were selected as they consume alcohol to intoxicating levels (> 80 mg/dL) without prior exposure [62, 65, 66, 67], while LAP mice provided an ideal comparison model due to their extremely low alcohol intake [52, 68], driven by similar genetic selection. We initially hypothesized that cHAP mice would exhibit higher levels of attention, motivation, and impulsivity compared to LAP mice, based on previous findings demonstrating that HAP1 and HAP2 mice, the progenitors of cHAPs, show increased impulsivity in delayed discounting tasks [52]. Contrary to our hypothesis, we found more evidence suggesting decreased impulsivity in the cHAP mice compared to the LAP, in addition to several other notable findings.

The most pronounced difference between the mouse lines was their response to more challenging task conditions. To ensure a comprehensive observation of the genetic alcohol preference on training, we included all mice, regardless of their performance levels. As training advanced, cHAPs were able to adapt effectively to shorter SDs. Conversely, LAPs exhibited a significant decline in correct responses and a marked increase in omission rates, particularly as the SD was reduced from 5 to 2 s and then to 1 s (Figure 1). When examining the reasons behind the LAP performance decline, we observed that their number of correct responses was approximately one‐third to one‐half of their cHAP counterparts (Figure 1). The overall accuracy of LAPs, while over ~75% during 2‐ and 1‐s SDs, indicated that they understood how to perform the task (Figure 1G,L). Although this was lower than that of cHAPs, it was well above chance (~20%) and met the standard 5‐Choice performance criterion [43]. We evaluated omission rates, which will increase in all mice as the SD decreases. Notably, LAP omission rates reached up to 50% (Figure 1H,M). To further understand this difference in performance, we analyzed correct latency (Figure 1N,S) and reward collection latency (Figure 1O,T). Correct latencies in LAPs were more variable and averaged 1 s longer than those of cHAPs. However, reward collection times remained under approximately 1.75 s across all groups, suggesting high motivation for the reward when it was presented. Based on these observations, we propose that the increased omission rates in LAPs were not due to a lack of interest in the reward or errors in attention, but rather a decreased drive to engage when faced with more challenging conditions. When returned to the less demanding five‐second baseline, LAP performance significantly improved toward response levels comparable to cHAP mice (indicated by 5 s‐B in Figure 1). This data may suggest that HAP mice are more genetically driven to overcome challenges for rewards. Conversely, LAP mice may have a reduced drive when faced with difficulty, potentially indicating a genetic inclination toward lower engagement under challenging conditions.

The most important caveat of this study is the differences in overall activity and energy expenditure, which may affect motivation and task engagement. Though this study does not specifically test these measures, there are several aspects of the study that suggest cHAP activity is significantly greater than LAP mice. For instance, experimenters found that traditional food restriction, where typically 2–2.5 g of chow would be sufficient to maintain body weight below 95%. This method was sufficient for LAP mice, but cHAP mice were found to be extremely lethargic within the first week of food restriction. Indeed, in order to maintain a healthy food restriction for cHAP mice required 3–4 g of chow. When comparing body weights during behavioral testing, cHAP mice weighed approximately 4% less than their ad libitum baseline compared to LAP mice (Figure S8). While this might suggest that LAP mice were less motivated than cHAPs, especially given their lower engagement levels, it is unlikely to fully explain their performance. LAP mice performed comparably to cHAPs during the 5 s‐T stage and showed higher engagement during the 2 s‐T sessions than at the 1 s‐B stage, when their body weights were slightly above *ad libitum* levels. During 5 s‐B, impulsivity, and attention testing, both cHAP and LAPs are below 95% *ad libitum*, though cHAP mice were significantly lower. However, a ~ 4% difference in body weight may not directly correlate with greater motivation, especially since cHAPs are more likely to be more lean considering their known activity patterns. First, experimenters noted that cHAP mice were generally more active in their homecage. Second, infrared video of cHAP versus LAP mice shows clear differences in methodology and energy given toward the 5‐Choice task (Video S1 and S2). Though we have qualitative data, previous studies offer some comparison. Xu et al. 2021 show that cHAP females have an average activity of ~7000 cm of distance traveled in a 15 min Open Field task [69]. In contrast, Can et al. 2011 found that LAP mice measure 5000 cm of distance traveled in 30 min of the same task [70]. Lastly, to compare both of these to the commonly used C57BL/6 mice, Shoji et al. 2016 found that distance traveled in 4–5‐month‐old mice was approximately 1000 cm in 5 min [71]. Together, it is clear that cHAP mice spend significantly more energy than LAPs and may be the driving reason behind their greater activity within the 5‐Choice. Importantly, the selective breeding of HAP has led to a hyperactivity trait that must be further explored. Together, we surmise that their motivation to perform the task was not the primary driver of the low performance in the LAPs.

There are other limitations to this study. One limitation of this study is the imbalance in sample sizes, with more cHAP mice than LAP mice. The smaller number of LAP mice contributes to greater variability in their data. Previous generations of LAP mice have demonstrated resistance to challenges, as evidenced by their higher susceptibility to developing conditioned taste aversion [72]. Therefore, we anticipated, and subsequently confirmed, that training these mice would be more difficult. Furthermore, because LAP mice are not typically the primary focus in alcohol studies due to their low interest in alcohol, our emphasis was on investigating potential sex differences within the cHAP line. Another limitation of the current study is that our interpretation of differences between cHAP and LAP mice must be made with caution. Frustration resulting from increased task difficulty may affect multiple outcome measures simultaneously. The cognitive‐behavioral engagement metrics we assessed, such as impulsivity, motivation, and attention, are not entirely independent, and the overall poorer performance of LAP mice, particularly at shorter SDs, may confound these interpretations. To mitigate this, we focused our comparisons on the 5 s‐B sessions, where LAP mice demonstrated more stable performance, reducing the likelihood of such confounding effects.

The 5‐Choice task used in this study is a well‐established behavioral paradigm that can assess waiting impulsivity, attention, perseveration, and motivation, making it a versatile task for collecting behavioral data [43, 48, 56, 73, 74, 75]. Impulsivity encompasses several behavioral constructs: 1) response inhibition, or the ability to withhold a prepotent response; 2) sensitivity to delayed consequences, or the devaluation of a consequence due to a time delay; 3) attention, the ability to focus on specific stimuli; and 4) risk sensitivity, or susceptibility to probabilistic decisions [76]. In this study, the 5‐Choice task specifically measured anticipatory response inhibition (the ability to wait for the stimulus) and attention. A premature response, touching a stimulus location before its presentation, delays the opportunity for a reward (premature responding), while accuracy in selecting the correct stimulus is required to obtain the reward (attention). In contrast, delay discounting tasks guarantee reward delivery but offer a smaller immediate reward versus a larger reward after a delay (delayed consequence sensitivity) [54, 77, 78, 79]. Our results suggest that cHAPs do not show increased waiting impulsivity compared to LAPs. However, given the impulsivity traits observed in parent HAP1 and HAP2 mouse lines in delay discounting [52], it is possible that their cHAP progeny may exhibit greater discounting behavior in future studies. Additionally, previous work in mice and rats has shown that innate impulsivity can be altered by various forms of alcohol administration. C57BL/6 inbred mice exposed to 4‐h alcohol vapor treatments exhibit increased innate impulsivity [75, 80]. Similarly, in rats, adolescent alcohol gavage and an alcohol‐enriched diet led to heightened impulsivity [81, 82]. A recent study outlined how to incorporate alcohol in the 5‐Choice task, showing that C57BL/6 mice self‐identify as higher alcohol‐preferring through homecage drinking and were more likely to exhibit greater engagement when seeking an alcohol reward within the task [83, 84]. Future studies involving the family history aspect of cHAP and LAP animals with alcohol treatment strategies and the 5‐Choice alcohol paradigm could reveal changes in engagement behaviors for sweet versus alcohol rewards.

This study revealed important sex differences within the cHAP line. First, cHAP females exhibited significantly greater perseveration during baseline, impulsivity, and attention sessions compared to cHAP males. In the LAP group, females also showed increased perseveration, but only during attention testing. Perseveration, defined as responses to the touchscreen after a correct or incorrect response has already been given, indicates cognitive inflexibility [46, 48, 60, 61, 85]. This behavior can reflect an inability to stop an action that is no longer rewarded or a failure in task‐switching [86] where, for example, a mouse should transition from making a correct response to retrieving the reward. Perseverative action has been observed to be greater in female rodents [87] and humans [88]. This metric may prove to be a vulnerability within females generally. For example, during a drinking session, a woman may continue seeking alcohol rather than switching to a nonalcoholic beverage, potentially leading to a binge drinking episode. This behavior reflects a form of cognitive inflexibility, where attention and decision‐making remain fixated on alcohol rather than transitioning to the next phase of the social outing. Second, cHAP females demonstrated significantly higher accuracy and lower correct latency in almost all behavioral conditions, suggesting they were more attentive in the task than their male counterparts. Notably, under the more challenging one‐second stimulus condition, the females responded within approximately 1 s, indicating their responses were nearly immediate. These findings, particularly the increased perseveration, raise important questions about sex differences in brain regions involved in cognitive‐behavioral processing, potentially driven by selective breeding for HAP. In particular, the salience network, which includes regions such as the anterior insula and anterior cingulate cortex, should be a focal point for future investigation [20, 89, 90, 91]. The anterior insula plays a critical role in detecting challenges and maintaining goal‐directed strategies in response, such as sustaining attentional vigilance during the waiting period in the 5‐Choice task [16, 92, 93, 94, 95]. For instance, anterior insular activation has been shown to strongly correlate with challenge‐based alcohol consumption [92]. Based on this, we speculate that neural activity in cHAP mice, especially females, would increase as task difficulty escalates, while LAP mice would show markedly lower activation. This would suggest that selective breeding for HAP enhances the ability to persist through cognitive challenge, a hypothesis further supported by the resistance of cHAP mice to quinine‐adulterated alcohol [30]. The anterior cingulate cortex, involved in decision‐making processes [96, 97, 98, 99, 100], is likely engaged at stimulus onset. The rapid response times observed in female cHAP mice may reflect swift cingulate activation to assess the correct stimulus and initiate a motor response, potentially more so than in male cHAPs. Exploring the neural synchrony between the anterior insula and anterior cingulate in male and female cHAPs could help explain the heightened task efficiency observed in females, an efficiency that may represent a behavioral vulnerability to alcohol and other drugs of abuse. Finally, while both the anterior insula and cingulate contribute to perseveration and behavioral inflexibility [101], these processes have also been extensively linked to the orbitofrontal cortex [85, 102]. Therefore, future studies should include investigation of these interconnected brain regions to better define the neural circuits underlying engagement and vulnerability, with the goal of informing targeted therapeutic interventions.

In summary, this study provides valuable insights into the behavioral characteristics of cHAP and LAP mice, highlighting the role of genetic predisposition in response to challenging tasks and the potential for sex differences in engagement and cognitive flexibility. While our original hypothesis that cHAP mice would display greater impulsivity and attentional differences was not supported, we identified significant sex differences within the cHAP line. These findings suggest that female cHAPs, with their higher accuracy, lower latency, and increased perseveration, may exhibit distinct neural activation patterns in regions related to cognitive processing, such as the anterior insula and anterior cingulate cortex. The unique responses observed under increasing task difficulty point to potential differences in how these brain regions contribute to behavioral regulation, particularly in females. Future research should integrate neural analyses to better understand the underlying circuitry associated with these behaviors and investigate how these sex and genetic differences might inform our understanding of predispositions to alcohol use and related disorders. Despite limitations such as sample size imbalance and variability in the LAP group, this study sets the stage for more targeted investigations into the interaction between genetic background, sex, and neural function in models of alcohol preference and behavior.

## Conflicts of Interest

The authors declare no conflicts of interest.

## Supporting information


**Data S1.** Supporting Information.

Table S1‐S5.

Video S1.

Video S2.

## Data Availability

The data that support the findings of this study are available from the corresponding author upon reasonable request.
