# Supplementary material for: Sex and Genetic Differences in Behavioral Engagement of Crossed High Alcohol‐Preferring and Low Alcohol‐Preferring Mice
Source: Genes Brain Behav. 2025 Jun 10;24(3):e70026. doi: 10.1111/gbb.70026 (PMC12150243; doi:10.1111/gbb.70026)
Supplement: Supplementary file 1 — Data S1. Supporting Information. [file GBB-24-e70026-s002.docx]

**Supplemental Material**

**Sex and Genetic Differences in Behavioral Engagement of Crossed High Alcohol-Preferring and Low Alcohol Preferring Mice**

Phillip Starski^1^, Addyson Siegle^1^, Danielle White^2^, Bea Paras^2^, Christy Tham^2^, MaribelHernandez^3^, Alecsander Zareb^1^, Nicholas Grahame^4^, Stephen L Boehm 2nd^4^, Frederic Hopf^1^

^1^Dept. Psychiatry, Indiana University School of Medicine, Indianapolis, IN, ^2^Dept. Psychology, IUPUI, Indianapolis, IN

Running Title: Alcohol Predisposition in 5-Choice Serial Reaction Time Task

*Correspondence: Dr. Phillip Starski, Department of Psychiatry, Indiana University School of Medicine, Stark Neuroscience Research Institute, 320 W 15th St., Indianapolis, IN 46202-2266, USA, Tel: (317)-278-5855, [pstarski@iu.edu](mailto:pstarski@iu.edu).

**Supplemental Results**

Supplemental Figure 1


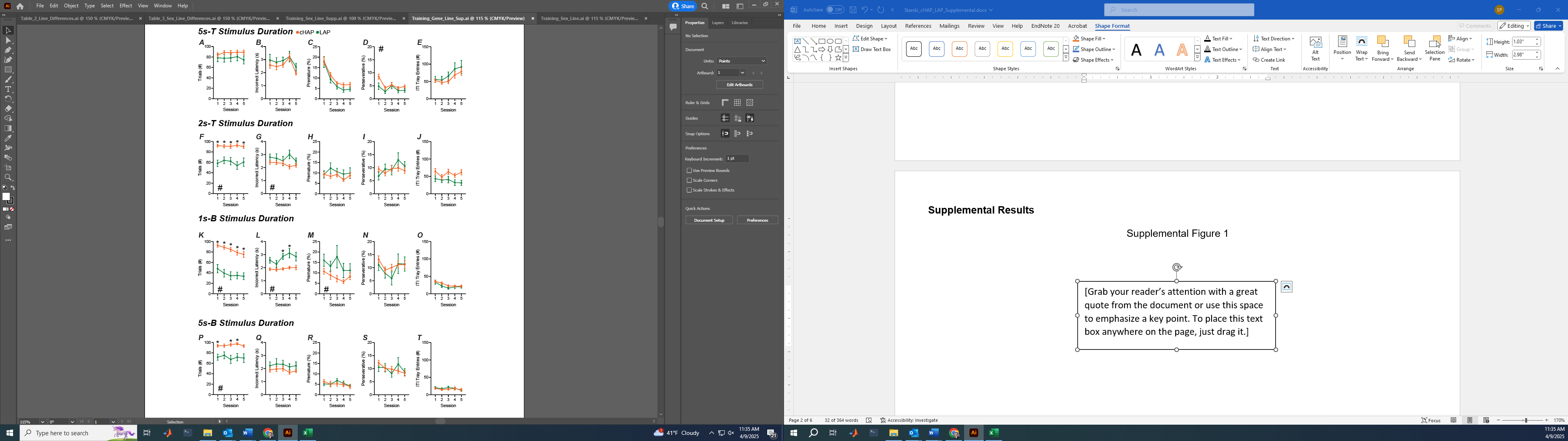


**Supplemental Figure 1. Secondary Measures of Line differences in training performance.** cHAP-male (orange) and LAP (green) lines were compared during the following stimulus training levels: five second training (5s-T), two second training (2s-T), one second baseline (1s-B), and the re-established five second baseline (5s-B). **A, F, K, P)** Number of started trials. **B, G, L, Q)** Time to give an incorrect response. **C, H, M, R)** Percentage of premature responses (premature/[premature+correct+incorrect)]*100). **D, I, N, S)** Perseverative percentage (perseverative responses/[perseverative responses+correct+incorrect]*100). **E, J, Q, T)** Number of head entries into the reward tray during the waiting period (intertrial interval, ITI). This analysis did not exclude any mice to demonstrate variability within the mouse lines. cHAP (n=20/sex), LAP (n=10/sex). Error bars represent standard error of the mean. **p<*0.05.

Supplemental Figure 2


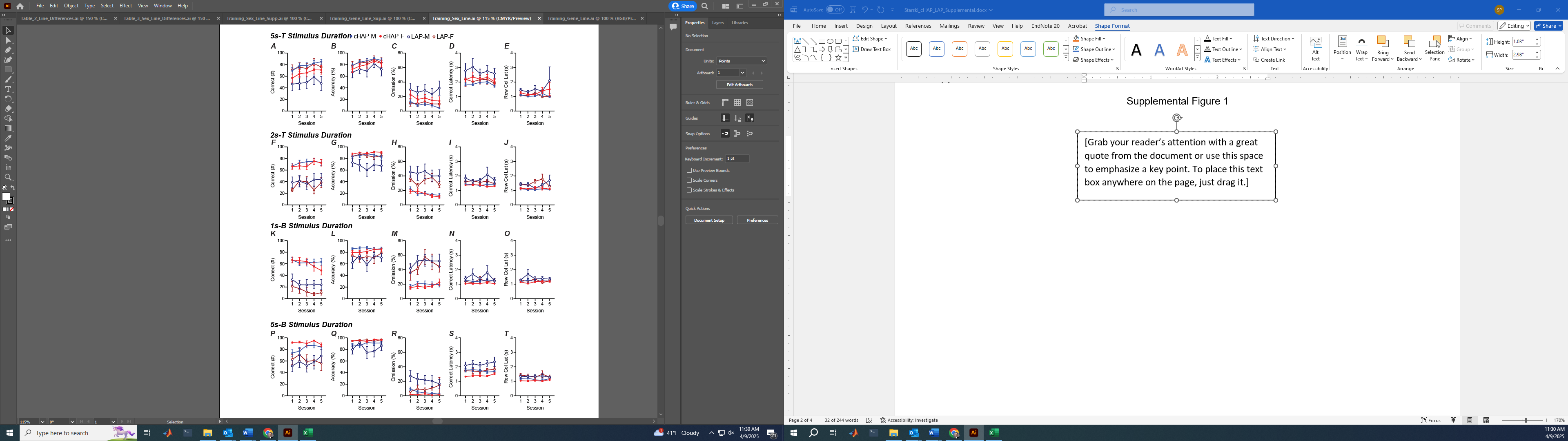


**Supplemental Figure 2. Training Performance Separated by Sex and Genetic Line.** cHAP-male (blue), cHAP-female (red), LAP-male (blue circle), and LAP-female (red circle) lines were compared during the following stimulus training levels: five second training (5s-T), two second training (2s-T), one second baseline (1s-B), and the re-established five second baseline (5s-B). **A, F, K, P)** Correct responses into the touch port at the respective stimulus. **B, G, L, Q)** Overall accuracy of responses (correct/[correct+incorrect]*100). **C, H, M, R)** Percentage of trial omissions (omitted trials/100*100). **D, I, N, S)** Time to give a correct response. **E, J, Q, T)** Time to retrieve the reward. This analysis did not exclude any mice to demonstrate variability within the mouse lines. cHAP (n=20/sex), LAP (n=10/sex). Error bars represent standard error of the mean. **p<*0.05.

Supplemental Figure 3


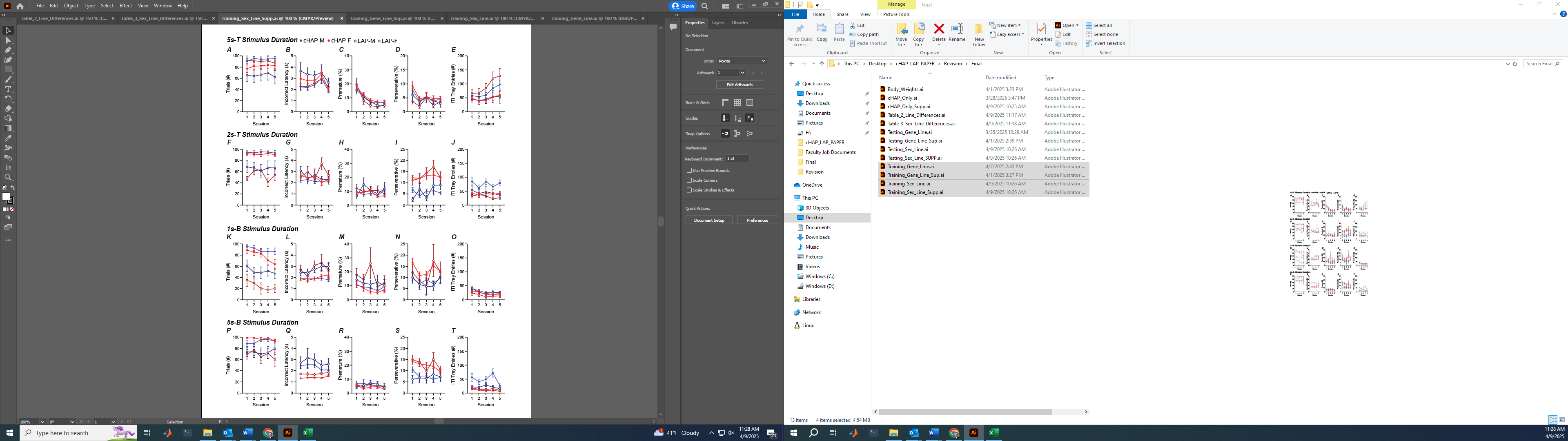


**Supplemental Figure 3. Secondary Measures of Training Performance Separated by Sex and Genetic Line.** cHAP-male (blue), cHAP-female (red), LAP-male (blue circle), and LAP-female (red circle) lines were compared during the following stimulus training levels: five second training (5s-T), two second training (2s-T), one second baseline (1s-B), and the re-established five second baseline (5s-B). **A, F, K, P)** Number of started trials. **B, G, L, Q)** Time to give an incorrect response. **C, H, M, R)** Percentage of premature responses (premature/[premature+correct+incorrect)]*100). **D, I, N, S)** Perseverative percentage (perseverative responses/[perseverative responses+correct+incorrect]*100). **E, J, Q, T)** Number of head entries into the reward tray during the waiting period (intertrial interval, ITI). This analysis did not exclude any mice to demonstrate variability within the mouse lines. cHAP (n=20/sex), LAP (n=10/sex). Error bars represent standard error of the mean. **p<*0.05.

Supplemental Figure 4


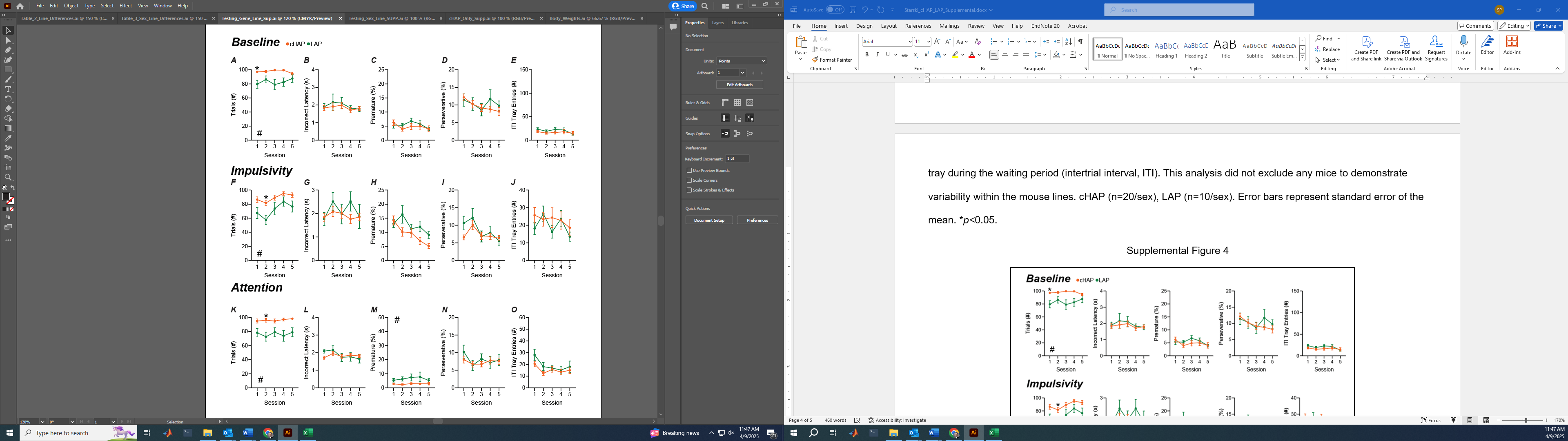


**Supplemental Figure 4. Secondary Behavioral Measures Across Genetic Lines During Testing.** cHAP (orange) and LAP (green) mice established a baseline performance under a 5s stimulus duration and 5s waiting period (Top). Impulsivity testing, where the waiting period was randomized (2, 5, 7, 10, 15s), was performed with a 5s stimulus duration (Middle). Attention testing was performed by randomizing the stimulus duration (0.5, 1, 1.5, 2, 2.5s) and having a static waiting period of 5s (Bottom). **A, F, K)** Number of initiated trials. **B, G, L)** Time to give an incorrect response. **C, H, M)** Premature response percentage. **D, I, N)** Perseverative response percentage. **E, J, O)** Head entries into the reward tray during the waiting period. Error bars represent standard error of the mean. **p<*0.05.

Supplemental Figure 5


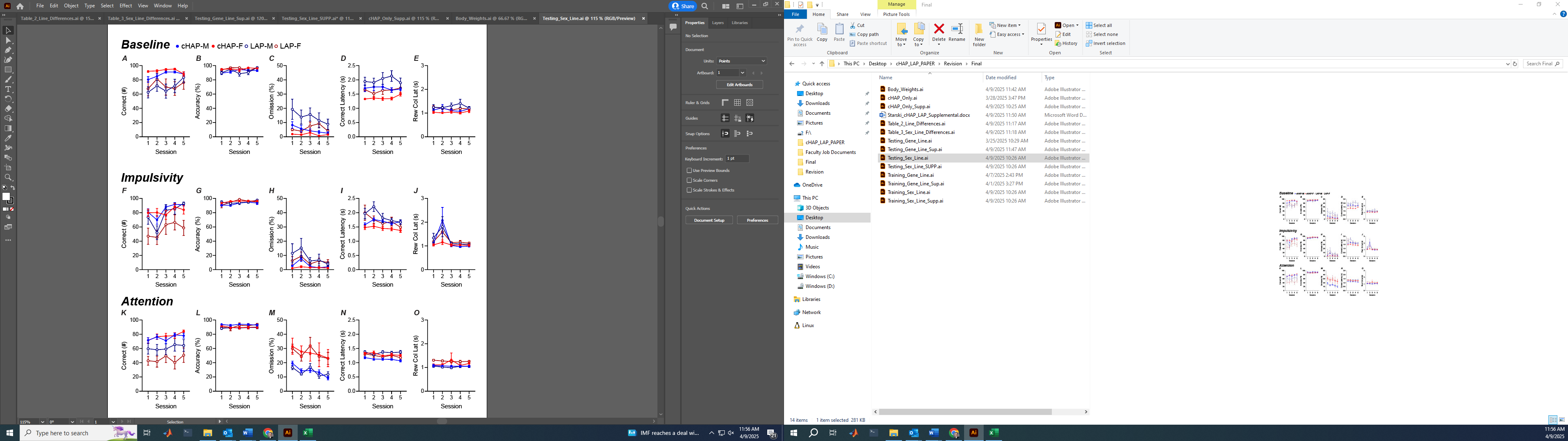


**Supplemental Figure 5. Behavioral Measures Across Sex and Genetic Lines During Testing.** cHAP-male (blue), cHAP-female (red), LAP-male (blue circle), and LAP-female (red circle) mice established a baseline performance under a 5s stimulus duration and 5s waiting period (Top). Impulsivity testing, where the waiting period was randomized (2, 5, 7, 10, 15s), was performed with a 5s stimulus duration (Middle). Attention testing was performed by randomizing the stimulus duration (0.5, 1, 1.5, 2, 2.5s) and having a static waiting period of 5s (Bottom). **A, F, K)** Correct Responses. **B, G, L)** Overall accuracy. **C, H, M)** Percentage of omissions. **D, I, N)** Time to give a correct response. **E, J, O)** Reward Collection Latency. Error bars represent standard error of the mean. **p<*0.05.

Supplemental Figure 6


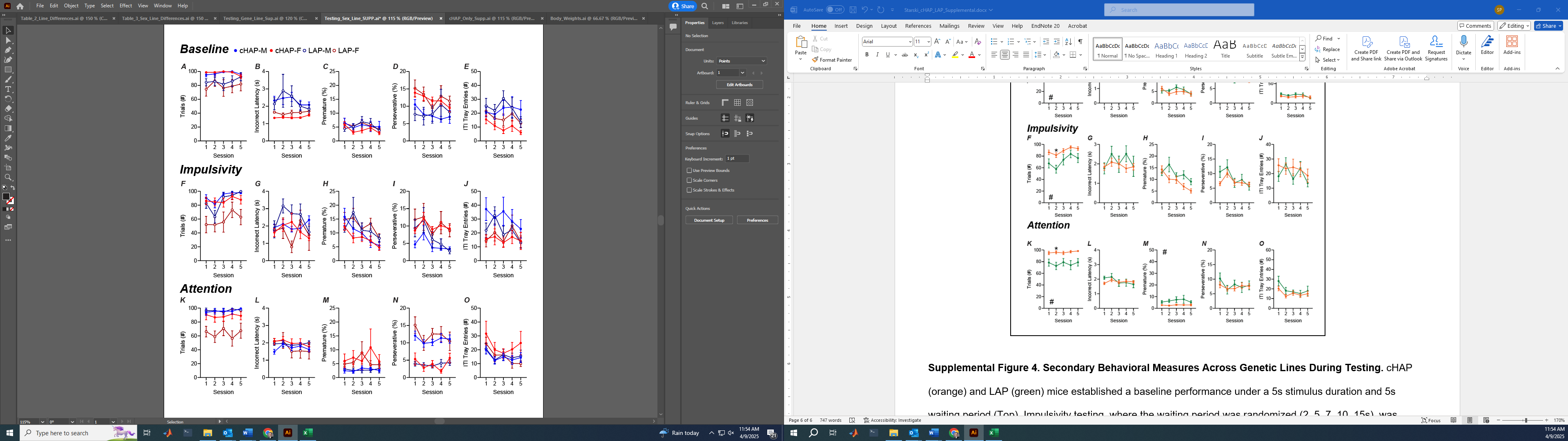


**Supplemental Figure 6. Secondary Behavioral Measures Across Sex and Genetic Lines During Testing.** cHAP (orange) and LAP (green) mice established a baseline performance under a 5s stimulus duration and 5s waiting period (Top). Impulsivity testing, where the waiting period was randomized (2, 5, 7, 10, 15s), was performed with a 5s stimulus duration (Middle). Attention testing was performed by randomizing the stimulus duration (0.5, 1, 1.5, 2, 2.5s) and having a static waiting period of 5s (Bottom). **A, F, K)** Number of initiated trials. **B, G, L)** Time to give an incorrect response. **C, H, M)** Premature response percentage. **D, I, N)** Perseverative response percentage. **E, J, O)** Head entries into the reward tray during the waiting period. Error bars represent standard error of the mean. **p<*0.05.

Supplemental Figure 7


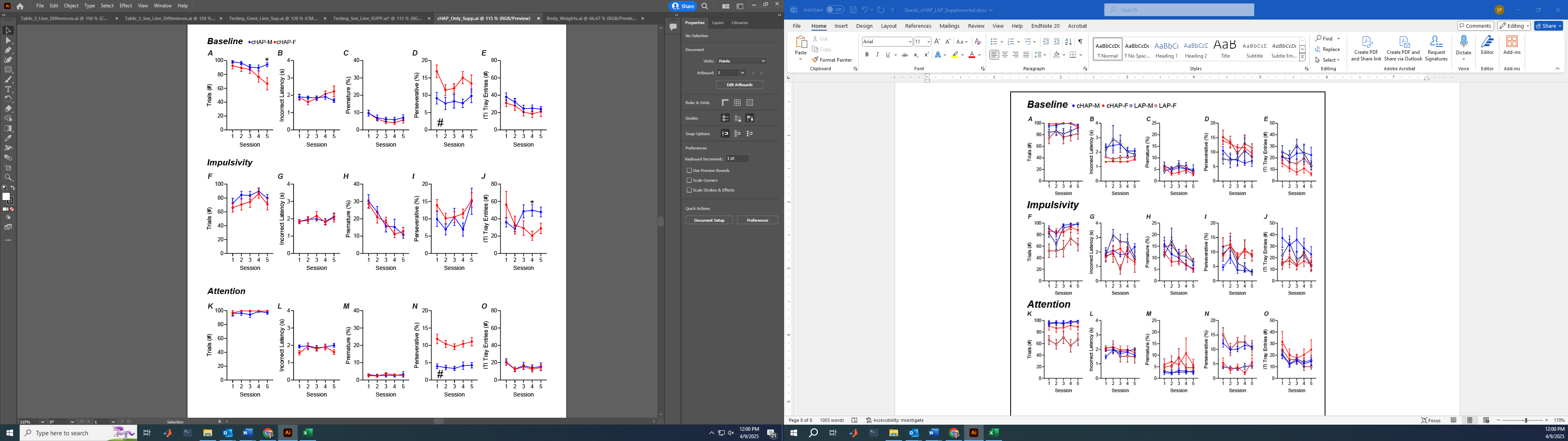


**Supplemental Figure 7. Secondary Behavioral Measures of cHAP Mice During 1s Testing.** cHAP-male (blue) and cHAP (red) mice established a baseline performance under a 1s stimulus duration and 5s waiting period (Top). Impulsivity testing, where the waiting period was randomized (2, 5, 7, 10, 15s), was performed with a 1s stimulus duration (Middle). Attention testing was performed by randomizing the stimulus duration (0.5, 1, 1.5, 2, 2.5s) and having a static waiting period of 5s (Bottom). **A, F, K)** Number of initiated trials. **B, G, L)** Time to give an incorrect response. **C, H, M)** Premature response percentage. **D, I, N)** Perseverative response percentage. **E, J, O)** Head entries into the reward tray during the waiting period. Error bars represent standard error of the mean. **p<*0.05.

Supplemental Figure 8


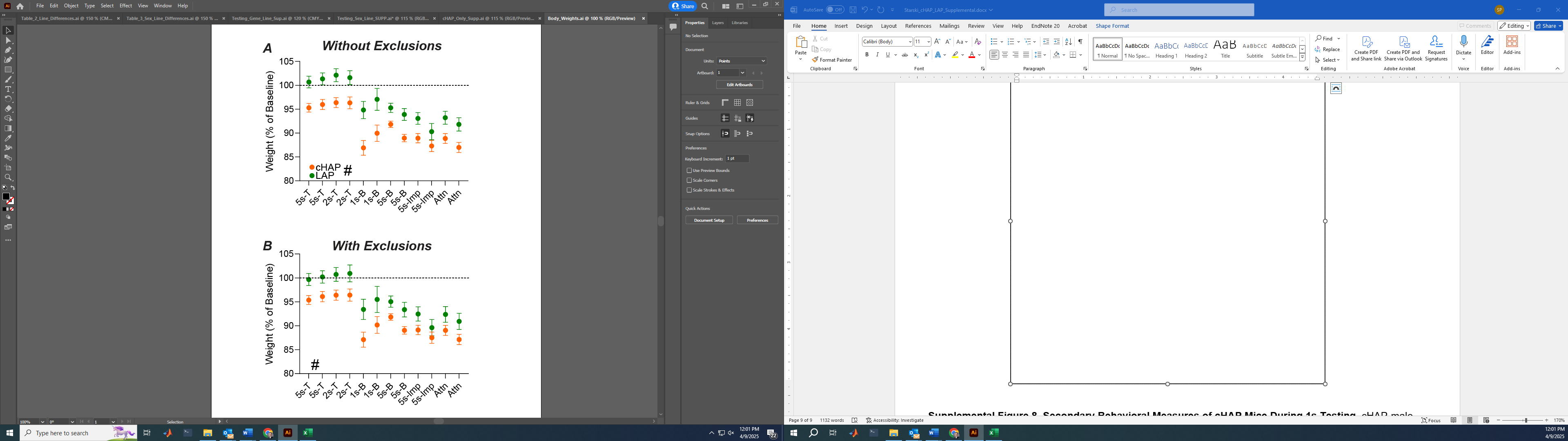


**Supplemental Figure 8. Body Weights of the cHAP and LAP Mice During Measured Training and Testing.** cHAP (orange) and LAP (green) mice were food restricted to promote responding in the 5-Choice. Weights were normalized to their *ad libitum* weight to standardize relative differences between the mice. cHAP mice weighed significantly lower than LAP mice without **(A)** or with **(B)** exclusions. Each timepoint represents the session 2 and 4 of each testing week. Error bars represent standard error of the mean. **p<*0.05.
